# The Practical Value of Porcelain as a Filling-Material

**Published:** 1902-04

**Authors:** William H. Potter


### Reviews of Dental Literature.

---

THE PRACTICAL VALUE OF PORCELAIN AS A FILLING-MATERIAL. By Alfr. Körbitz, Berlin.[1]

The æsthetic value of porcelain fillings is commonly admitted. There are, however, other points to be considered. One of these is that the practice of "extension for prevention" can better be carried out by the use of porcelain fillings than by the use of gold or any of the plastics. This is possible because the loss of substance resulting from the free cutting away of the walls of the cavity is readily supplied by a material which closely resembles tooth-substance, and makes its loss unobserved. As to the durability of

---

[1] Der praktische Werth des Porzellans als Füllungsmaterial, von Alfr. Körbitz, Zahnarzt in Berlin. Deutsche Monatsschrift für Zahnheilkunde, 14. Dezember, 1901.

porcelain fillings, the author has these important words to say: "A porcelain filling holds so long as it preserves the tooth. If a filling falls out when it no longer serves its purpose, its importance as a preservative filling is so much heightened. If all gold, amalgam, and cement fillings, about which secondary caries exists, would fall out, the affected teeth would be better served than by their remaining hanging in undercuts and protecting and concealing the carious area. Porcelain fillings must, on account of the way in which they are inserted, soon lose their hold when secondary decay penetrates the depths of the cavity. The thin line of cement which exists about a well-executed porcelain filling is not, in the case of normally formed teeth, a place of less resistance, since the edges of enamel which may be exposed by the dissolving of cement are as capable of withstanding caries as the smooth superficial surfaces of the teeth. It is worthy of notice that the ability of cement under these circumstances to resist deterioration has given rise to many attempts at an explanation."

The author concludes that, from the evidence already at hand, the fact is established that porcelain fillings stand high as preservers of tooth-substance.

That porcelain fillings sometimes fall out prematurely cannot, however, be denied. And the reasons for this are given as follows: 1. A faulty execution of the work as regards depth of cavity, fit of the margin, undercut, consistency of cement, or articulation. 2. An unfavorable reaction of the fluids of the mouth.

As to what cavities are suitable for porcelain fillings, the author quotes Professor Miller, as follows: "Porcelain fillings can be used in all places where a good impression can be made."

A practical consideration of the preparation of cavities follows, for on this depends the success or failure of the filling. The general shape of the cavity is that of a trough with steep walls which broaden outward in a funnel-shaped way. The taking of the impression is stated as the most difficult part of the work. The author uses Williams's rolled gold No. 30 for the matrix, and says that in the unheated state it takes the best impression, but is also in this state less liable to resist change of form in removal. By heating, the elasticity of the gold is increased, and it holds more tenaciously to a given form, but it is also less pliable.

In regard to instruments, the author recommends a pair of pliers much heavier than the ordinary foil-pliers, and one having each point finished with a half-sphere piece of metal. The two half-spheres come together on their flat surfaces with the closing of the pliers. With this form of pliers a piece of cotton or other material can be forced against the matrix with the minimum danger of tearing it. Chamois-skin leather and sponge are recommended for pressing the gold matrix into place.

There are three mistakes which can be made in the taking of the impression. First, the piece of foil can be so large as to overlap the cavity to such an extent as to make the withdrawing of the matrix difficult or impossible. Second, the endeavor can be made to take an exact impression of the bottom of the cavity. This is undesirable, for a certain amount of room is needed in the bottom of the cavity for cement; moreover, the bottom of the cavity is often rough and likely to hinder the withdrawing of the matrix if closely fitting it. Third, the use of ball-burnishers to burnish the gold against the walls of the cavity.

William H. Potter.

